# Microfilaremic infection in canine filariosis in Colombia: a challenge in morphological and molecular diagnostics

**DOI:** 10.3389/fvets.2024.1368307

**Published:** 2024-03-27

**Authors:** María Victoria Esteban-Mendoza, Victor Hernán Arcila-Quiceno, Catalina Ríos Chacón, Jeiczon Elim Jaimes Dueñez, Marisol Tique Oviedo, Alejandro Díaz Bustos, María Fernanda Castellanos, Rodrigo Morchón

**Affiliations:** ^1^Grupo GRICA, Facultad de Medicina Veterinaria y Zootecnia, Maestría en Salud y Producción Animal, Universidad Cooperativa de Colombia, Bucaramanga, Santander, Colombia; ^2^Biovet Diagnóstico Veterinario Bga, Laboratorio Clínico Veterinario, Floridablanca, Santander, Colombia; ^3^Zoonotic Diseases and One Health GIR, Biomedical Research Institute of Salamanca-Research Center for Tropical Diseases University of Salamanca (IBSAL), Faculty of Pharmacy, Campus Miguel Unamuno, University of Salamanca, Salamanca, Spain; ^4^Centre for Environmental Studies and Rural Dynamization (CEADIR), University of Salamanca, Salamanca, Spain

**Keywords:** filariosis, zoonosis, Colombia, dogs, *Dirofilaria immitis*, *Acanthocheilonema reconditum*

## Abstract

Canine filariosis is caused by filiform nematodes and affects several species of animals as well as humans. The disease produces a wide range of symptoms that can often be confused with other diseases, which increases the complexity of its diagnosis. The search for methodologies to facilitate its diagnosis is a challenge, and specific and differential identification of the parasite species causing the disease holds key to a successful diagnosis. In Colombia, there is a problem of underdiagnosis of filariosis in microfilaremic dogs infected by *Dirofilaria immitis* and *Acanthocheilonema reconditum*, and of microfilaremias not related to heartworm disease. The highest prevalences have been reported for *D. immitis* infections, although new cases of *A. reconditum* infections are beginning to appear. The aim of this study was to differentiate the microfilariae infections caused by *D. immitis* and *A. reconditum* by a morphological and molecular characterization of microfilariae so as to facilitate an accurate diagnosis of canine filariosis in the metropolitan area of Bucaramanga (Colombia). For this purpose, 400 blood samples with anticoagulants were collected from the dogs and analyzed with the help of a commercial immunochromatography kit for the detection of *D. immitis* circulating antigen. The Woo, Knott, and polymerase chain reaction (PCR) techniques were employed for determining the parasite count, morphological observation, and molecular identification of microfilariae present in the dogs respectively. The prevalence of microfilaremic dogs in Bucaramanga metropolitan area was 18.75% (75/400). The prevalence of dogs that tested positive for *D. immitis* in the antigen and in PCR tests was 1.25% (5/400) and 1% (4/400), respectively. Furthermore, the PCR test revealed that 17.75% of the microfilaremic dogs tested positive for *A. reconditum* (71/400) (first report in the metropolitan area of Bucaramanga), with one animal co-infected by both species, and 0% for *D. repens* (0/400). However, by morphological characterization, 4% of the microfilariae (3/75) corresponded to *D. immitis*, 20% (15/75) to *D. repens*, and 76% (57/75) to *A. reconditum*. The use of molecular diagnostic methods such as PCR aids in the specific identification of the parasite, thus making it a more accurate method than the morphological characterization of microfilariae. The identification of the parasites by PCR helps improve the veterinary diagnosis of canine filariosis in Colombia, which would lead to the establishment of an appropriate treatment protocol for each species of filaria and also to the generation of reliable data to be used at the clinical and epidemiological levels.

## 1 Introduction

Canine filariosis is a parasitic disease caused by filiform nematodes at different stages of their life cycle (1). These parasites are transmitted by vectors that are widely distributed, with the main host being dogs, both domestic and wild, as well as humans, who act as accidental hosts (2). In veterinary medicine, there are families of filarials that hold importance for their impact on public health due to their zoonotic potential, although filarials such as *Onchocerca lupi, Acanthocheilonema dracunculoides (sin: Dipetalonema dracunculoides), A. reconditum (sin: Dipetalonema reconditum),* and *Cercopithifilaria (sin: Acanthocheilonema grassi)* (1, 3–7) cause low pathogenicity and other pathogens such as *Dirofilaria immitis* and *D. repens* are responsible for heartworm disease and subcutaneous dirofilariosis, respectively (8, 9).

These diseases produce different clinical manifestations. On the one hand, they may lead to ataxia, incoordination, marked leukocytosis, and hemoglobinuria, caused by an infection by *A. dracunculoidess*, which are lodged in the peritoneal cavity (8, 10, 11). Granulomas formed at the cutaneous level due to the presence of the parasite in muscle fascia, subcutaneous tissue, peritoneal cavity, and kidney are associated with an infection by *A. reconditum* (12, 13). On the other hand, *D. immitis* can cause chronic cough, dyspnea, lipothymia, weakness, anorexia, weight loss, and dehydration, depending on the parasite load or variation in the physical exercise performed by the animal. The greater the physical activity, the greater the arterial damage (9). In addition, the symptoms caused by *D. repens* depend on the location of the nodules it infects and are generally limited to local inflammation, mainly in subcutaneous and ocular tissues, erythema, and pruritus. Occasionally, much more severe systemic immune reactions may develop, manifesting as fever or lymphadenopathy (14, 15).

Different vector species are involved in the transmission of these parasites. *Rhipicephalus sanguineus* (tick), *Ctenocephalides* spp., and *Pulex irritans* (fleas) are the vectors known to transmit *A. dracunculoidess* and *A. reconditum* (7), whereas *Culex* spp., *Aedes* spp., and *Anopheles* spp. (culicid mosquitoes) are implicated in the transmission of *D. immitis* and *D. repens* (16).

A wide variety of studies have reported the prevalence of *D. immitis* worldwide in dogs, mainly due to the availability of commercial diagnostic techniques for the detection of circulating antigens (9, 16–18). However, for other species, cases are reported accidentally, epidemiological studies are rare, and there are no commercial diagnostic techniques available for their detection (13, 19–21). In Colombia, the prevalence of *D. immitis* in dogs is 0.91–53.2%, depending on the sampling site (22–27), and it is 10.82% in Bucaramanga (28). However, there is only one study that has reported the presence of *D. repens* using molecular techniques in three blood samples of dogs that are found in shelters in Bucaramanga (29). There are two reports on the presence of *A. reconditum* in microfilaremic dogs with prevalences varying between 4.81 and 61.3% (13, 30).

This complex diagnostic condition and the fact that it is common to observe dogs with a myriad of symptoms that can be associated with various diseases makes the identification, differentiation, and diagnosis of filarial species at the veterinary clinic level very important. Therefore, the aim of this study was to provide techniques for the morphological and molecular characterization of the filariae present in dogs in the metropolitan area of Bucaramanga, Colombia, in order to improve the identification of filarial species and also contribute to their detection in other Colombian regions.

## 2 Materials and methods

### 2.1 Sampling area

**Figure 1:**
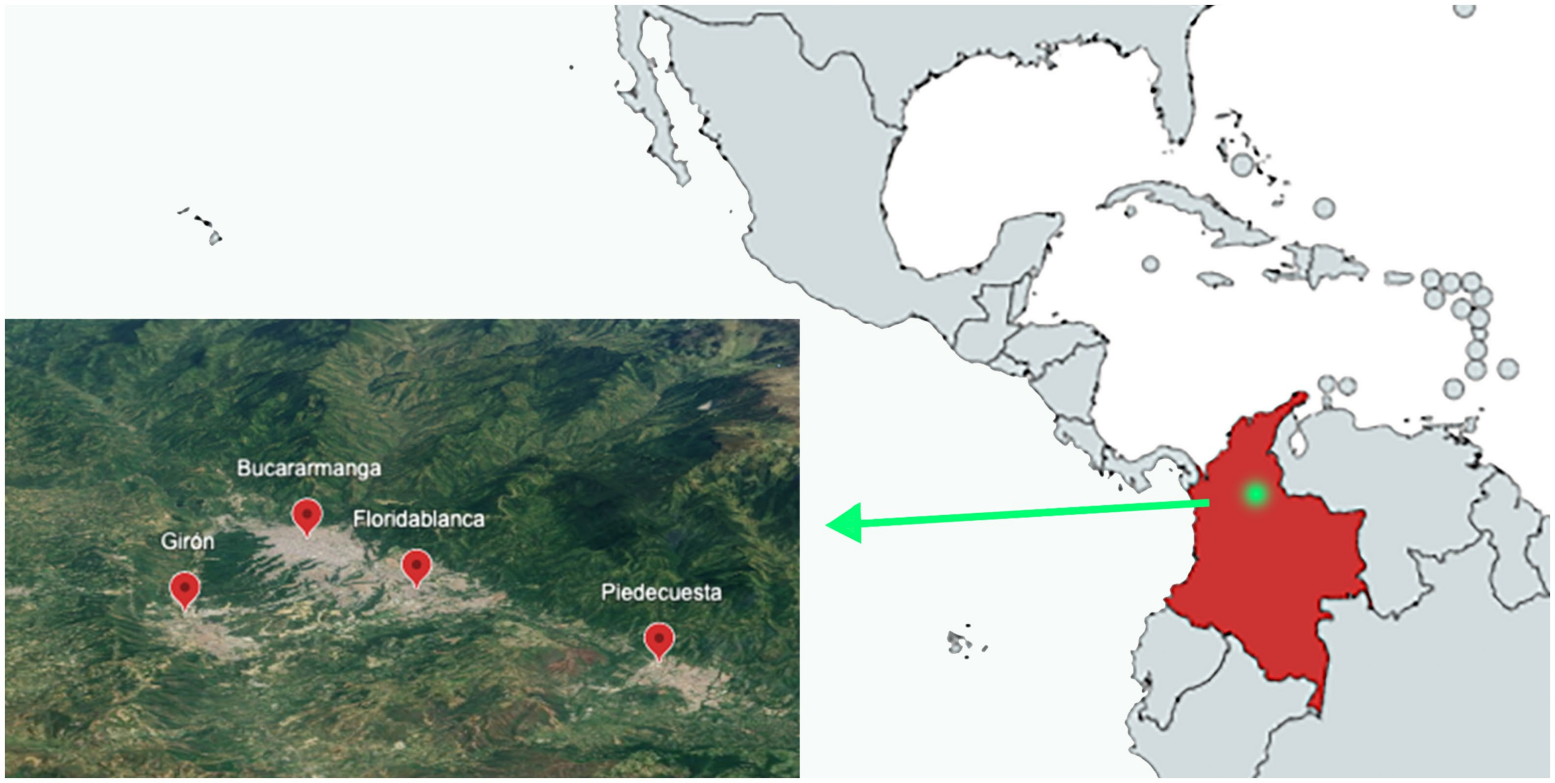
Sampling area: metropolitan area of Bucaramanga (Colombia) by satellite image.

Bucaramanga Metropolitan Area, the capital city of the Department of Santander, consists of three municipalities: Floridablanca, Piedecuesta, and Girón in central Colombia (Figure 1). This metropolitan area is spread over 1,479 km^2^, and its municipal area occupies 165 km^2^. It is located 959 m above the mean sea level. Its climate is tropical with an average temperature of 23°C and a maximum of 30°C, and the area experiences rainy and dry seasons throughout the year. The region receives significant annual rainfall at an average of 1,159 mm (31, 32). The estimated human population of the metropolitan area of Bucaramanga is 1.1 million, out of which 95% live in urban areas (33). The approximate domestic canine population is 7,906, according to the latest canine census of 2018–2019, of which a large number are stray dogs (34, 35).

### 2.2 Samples used

The samples collected from 400 owned domestic dogs (non-roaming) from January to September 2023 were used for this study. The veterinary staff members of the Biovet Veterinary Clinical Laboratory collected the samples. The written informed consent of the owners was considered as an inclusion criterion, and confidentiality of patient information was always maintained. All dogs included in the study were over 1 year of age. The variables considered for the analysis were age, sex, municipality of residence, socioeconomic status, whether dogs lived inside or outside of the house, and use or non-use of ectoparasite medication. The socioeconomic classification of the dogs was made based on the six socioeconomic hierarchical strata: (1) misery; (2) poverty; (3) poverty with some economic resources (4) middle class; (5) upper middle class; and (6) upper class. They were recategorized into vulnerable strata 1 and 2; middle class 3 and 4; and upper class 5 and 6 (36). All data are shown in Table 1.

**Table 1:** Identified sequences and GenBank accession number.

**Sample ID**	**Species filaria**	**Gene**	**Query coverage (%)**	**Sequence**	**GenBank number**
4	*Acanthocheilonema reconditum*	12S ribosomal RNA	94	yATTCGGGAGTAAAGTTGTATTTAAACCGAAAAAATATTGACTGACTTTAGATTTTTCTTTGGAATATGTGTTAGGAGAGCCCTCCTTATTTGTTTAATTTTTTTTGGCACATGTATGATTGTTTTGTTATTATGTTATTTGTAATGCTTTAAACTTTTTTTGTTTTAAAACAGATATATATTTGGCTTATAGATTTTTTTTGCATGTATTACTATTGTTAATTTTCTTTGGATATTTTTTTTATTTTTTTTTGAAATTGGAAAAGAAAGTAATTTATTTTTTAGTGTTTTAGTGAATTTAATAAATAGAGTGGTACAAACCTCCCCGTCAATT	PP214446
6	*Acanthocheilonema reconditum*	12S ribosomal RNA	89	CGATAATACYTRCCATAATATCATGATMTGWGTATTYTATTTTTYWATWTWATWTWTGTAAATATTTTAATTTTTTATTTTTAATTGAATAAATGTTTAAAATTTGTTTTGTGAACTGGATTAGTACCCAGGTAATCAAAGTTTATTAATTCGGGAGTAAAGTTGTATTTAAACCGAAAAAATATTGACTGACTTTAGATTTTTCTTTGGAATATGTGTTAGGAGAGCCCTCCTTATTTGTTTAATTTTTTTTGGCACATGTATGATTGTTTTGTTATTATGTTATYTGTAATGCTTTAAACTTTTTTTGTTTTAAACAGATATATATTTGGCTTATAGATTTTTTTTGCATGTATTACTATTGTTAATTTTCTTTGGATATTTTTTTATTTTTTTTGAAWTGSAAAAGAAGWATTTWTTTTTAGTGTTTAGWGATTTATAATAGAGYGYACMACCMTCCGTCATA	PP214447
9	*Acanthocheilonema reconditum*	12S ribosomal RNA	93	GCGTATACTCATCCGTACATACGTTATTTTTGTGTTTTTTATTTTTTTATTTTATTTTTGTAAAATATTTTAATTTTTTATTTTTAATTGAATAAATGTTTAAAATTTGTTTTGTGAACTGGATTAGTACCCAGGTAATCAAAGTTTATTAATTCGGGAGTAAAGTTGTATTTAAACCGAAAAAATATTGACTGACTTTAGATTTTTCTTTGGAATATGTGTTAGGAGAGCCCTCCTTATTTGTTTAATTTTTTTTGGCACATGTATGATTGTTTTGTTATTATGTTATTTGTAATGCTTTAAACTTTTTTTGTTTTAAAACAGATATATATTTGGCTTATAGATTTTTTTTGCATGTATTACTATTGTTAATTTTCTTTGGATATTTTTTTTATTTTTTTTTGAAATTGGAAAAGAAAGTAATTTATTTTTTAGTGTTTTAGTGAATTTAATAAATAGAGTGGTACAAAATTC	PP214448
11	*Dirofilaria immitis*	12S ribosomal RNA	95	TGGATTACTCTCTTTCGTGTACATTCTTACGATTTTTTTTGTTTTTTTGTTTTATGTTTTTTTTTGTAAAATATTTAAATTTATTTATGTTTTTTTGTAATATTGAAAATTTGGTGTTTGAACTGGATTAGTACCCAGGTAATCAAAATTTATTAATTCGGGAGTAAAGTTTTGTTTAAACCGAAAAAATATTGACTGACTTTAGATTTTTCTTTGGAATATGTGTTTTTTTGGAGAGCCCTCTTTTATAGTGAATTTTGTTGGCGCATGTATGATTGTTTAGTTTTTACTTTTTTGGTAATGCTTTGTGTTTTATACATTTAAAACAGATATATATTTGGCTTATGGATTTATTTTTCATGTGTTACTATTGTTAATTTTCTTTGGATTAATTTTTAATTTTTTTGTTGAAATTGGAAAAGAAAGTAATTTTTTCTTAATGTAATAATGAATTTAATAAATAAAGTGGTACAAATCCCACCCTAAAAG	PP158631:32
12	*Acanthocheilonema reconditum*	12S ribosomal RNA	80	CTCCATATMCTGCCAGCACATSAYTAYATMTGTSTGTTYTAYTTATTCTAYYKTATWTATGTAATATTTTAATTTTTTATTTTTAATTGAATAATGTTTAAATTTGTTTTGTGACTGGATTAGTACCCAGGTAATCAAAGTTTATTAATTCGGGAGTAAGTTGTATTTAAACCGAAAATATTGACTGACTTTAGATTTTTCTTTGGATATGTGTTAGGAGAGCCCTCCTTATTTGTTAATTTTTTTTGGCACATGTATGATGTTTTGTTATTATGTTATTTGTAATGCTTTAACTTTTTTTGTTTAAACAGATATATATTTGGCTTATAGATTTTTTTGCTGTATTACTATWGWTAATTTTCTTTGGTATTTTTTTATTTTTTTGAAATRSAAARAAGWATTYATTTTTAGTGTTTTAGWGAATTAKAATMSMSCGCCCCACCMTCCGTCATA	PP214449
13	*Acanthocheilonema reconditum*	12S ribosomal RNA	93	AGGCAGCTCTCCTTCTTACAAGCGATAATTTTAGTGTTTTTTATTTTTTTATTTTATTTTTGTAAAATATTTTAATTTTTTATTTTTAATTGAATAAATGTTTAAAATTTGTTTTGTGAACTGGATTAGTACCCAGGTAATCAAAGTTTATTAATTCGGGAGTAAAGTTGTATTTAAACCGAAAAAATATTGACTGACTTTAGATTTTTCTTTGGAATATGTGTTAGGAGAGCCCTCCTTATTTGTTTAATTTTTTTTGGCACATGTATGATTGTTTTGTTATTATGTTATTTGTAATGCTTTAAACTTTTTTTGTTTTAAAACAGATATATATTTGGCTTATAGATTTTTTTTGCATGTATTACTATTGTTAATTTTCTTTGGATATTTTTTTTATTTTTTTTTGAAATTGGAAAAGAAAGTAATTTATTTTTTAGTGTTTTAGTGAATTTAATAAATAGAGTGGTACAAACCCCCCCTCAAAAAA	PP214450
14	*Acanthocheilonema reconditum*	12S ribosomal RNA	92	AAACGTACTATCATTCGCTGCAATATTTATTTTTAGTGTTTTTTATTTTTTTATTTTATTTTTGTAAAATATTTTAATTTTTTATTTTTAATTGAATAAATGTTTAAAATTTGTTTTGTGAACTGGATTAGTACCCAGGTAATCAAAGTTTATTAATTCGGGAGTAAAGTTGTATTTAAACCGAAAAAATATTGACTGACTTTAGATTTTTCTTTGGAATATGTGTTAGGAGAGCCCTCCTTATTTGTTTAATTTTTTTTTGGCCCATGTATGATTGTTTTGTTATTATGTTATTTGTAATGCTTTAAACTTTTTTTGTTTTAAAACAAATATATATTTGGCTTATAAATTTTTTTTGCATGTATTACTATTGTTAATTTTCTTTGGATATTTTTTTTATTTTTTTTTGAAATTGAAAAAAAAAGTAATTTATTTTTTAGGGTTTTAGGGAATTTAATAAATAAAGGGGTCCAAACCCCCCCCCCAATAA	PP214451
15	*Acanthocheilonema reconditum*	12S ribosomal RNA	91	GGGAGACGATATTTAGTTTCTACGTTATTTTTGTGTTTTTTATTTTTTTATTTTATTTTTGTAAAATATTTTAATTTTTTATTTTTAATTGAATAAATGTTTAAAATTTGTTTTGTGAACTGGATTAGTACCCAGGTAATCAAAGTTTATTAATTCGGGAGTAAAGTTGTATTTAAACCGAAAAAATATTGACTGACTTTAGATTTTTCTTTGGAATATGTGTTAGGAGAGCCCTCCTTATTTGTTTAATTTTTTTTTGGCACATGTATGATTGTTTTGTTATTATGTTATTTGTAATGCTTTAAACTTTTTTTGTTTTAAAACAGATATATATTTGGCTTATAGATTTTTTTTGCATGTATTACTATTGTTAATTTTCTTTGGATATTTTTTTTATTTTTTTTTGAAATTGGAAAAAAAAGTAATTTATTTTTTAGGGTTTTAGTGAATTTAATAAATAGAGGGGTACAAACTCCCCCCCCAATATTAA	PP214452
16	*Acanthocheilonema reconditum*	12S ribosomal RNA	100	AGGTAATCAAAGTTTATTAATTCGGGAGTAAAGTTGTATTTAAACCGAAAAAATATTGACTGACTTTAGATTTTTCTTTGGAATATGTGTTAGGAGAGCCCTCCTTATTTGTTTAATTTTTTTTGGCACATGTATGATTGTTTTGTTATTATGTTA	PP214453
18	*Acanthocheilonema reconditum*	12S ribosomal RNA	89	TKKTCMTATWTTWATWWTATWTWGTAAATATTTTAATTTTTTATTTTTATTGRATAAATGTTTAAAATTTGTTTTGTGAACTGGATTAGTACCCAGGTAATCAAAGTTTATTAATTCGGGAGTAAAGTGTATTTAAACCGAAAAATATTGACTGACTTTAGATTTTTCTTTGGAATATGTGTTAGGAGAGCCCTCCTTATTTGTTAATTTTTTTTGGCACATGTATGATGTTTTGTATTATGTTA	PP214454
19	*Acanthocheilonema reconditum*	12S ribosomal RNA	100	ATATTTTAATTTTTTATTTTTAATTGAATAAATGTTTAAAATTTGTTTTGTGAACTGGATTAGTACCCAGGTAATCAAAGTTTATTAATTCGGGAGTAAAGTTGTATTTAAACCGAAAAAATATTGACTGACTTTAGATTTTTCTTTGGAATATGTGTTAGGAGAGCCCTCCTTATTTGTTTAATTTTTTTTGGCACATGTATGATTGTTTTGTTATTATGTTATTTGTAATGCTTTAAACTTTTTTTGTTTTAAAACAGATATATATTTGGCTTATAGATTTTTTTTGCATGTATTACTATTGKTAA	PP214456
26	*Acanthocheilonema reconditum*	12S ribosomal RNA	99	AATATTTTAATTTTTTATTTTTAATYGAATAAAAGKTTTAAAATTTGTTTTGKGAACTGGGATTAGTACCCCRGTAATCAAAGTTTWTTAATTCGGGAGTAAAGTTGTATTTAAACCGAAAAAATATTGACTGACTTTAGATTTTTCTTTGGRATATGTGTTAGGAGAGCCCYCCTTAWTTGTTTAATTTTTTTTGGCACATGTATGATTGTTTTGTTATTATGTTATTTGTAATGCTTTAAACTTTTTTTGTTTTAAAACAGATATATATTTGGCTTATAGATTTTTTTTGCATGTATTACTATTGTTATT	PP214457
27	*Acanthocheilonema reconditum*	12S ribosomal RNA	94	GCCATGACTATCCTTCCATACTTGTTTATTTTTAGTGTTTTTTATTTTTTTATTTTATTTTTGTAAAATATTTTAATTTTTTATTTTTAATTGAATAAATGTTTAAAATTTGTTTTGTGAACTGGATTAGTACCCAGGTAATCAAAGTTTATTAATTCGGGAGTAAAGTTGTATTTAAACCGAAAAAATATTGACTGACTTTAGATTTTTCTTTGGAATATGTGTTAGGAGAGCCCTCCTTATTTGTTTAATTTTTTTTTGGCACATGTATGATTGTTTTGTTATTATGTTATTTGTAATGCTTTAAACTTTTTTTGTTTTAAAACAGATATATATTTGGCTTATAGATTTTTTTTGCATGTATTACTATTGTTAATTTTCTTTGGATATTTTTTTTATTTTTTTTTGAAATTGGAAAAAAAAGTAATTTATTTTTTAGTGTTTTAGTGAATTTAATAAATAGAGTGGTACAAACCCCCCCCGTCAATAA	PP214458
28	*Acanthocheilonema reconditum*	12S ribosomal RNA	100	TTATTTTTGTGTTTTTTATTTTTTATTTATTTTWGKAAAATATTTTAATTTTTTATTTTTAATTGAATAAATGTTTAAAATTTGTTTTGTGAACTGGATTAGKACCCAGGTAATCAAAGTTTATTAATTCGGGAGTAAAGTTGTATTTAAACCGAAAAAATATTGACTGACTTTAGATTTTTCTTTGGAATATGTGTTAGGAGAGCCCTCCTTATTTGTTTAATTTTTTTTGGCACATGTATGATTGTTTTGTTATTATGTTATTTGTAATGCTTTAAACTTTTTTTGTTTTAAAACAGATATATATTTGGCTTATAGATTTTTTTTGCATGTATTWCTATTGTTAATTTTCTTTG	PP214459
30	*Acanthocheilonema reconditum*	12S ribosomal RNA	91	GGCAGGACKGAAACCTAAGAYARTTACTTTCTTTTCCAATTTCAAAAAAAAATAAAAAAAATATCCAAAGAAAATTAACAATAGTAATACATGCAAAAAAAATCTATAAGCCAAATATATATCTGTTTTAAAACAAAAAAAGTTTAAAGCATTACAAATAACATAATAACAAAACAATCATACATGTGCCAAAAAAAATTAAACAAATAAGGAGGGCTCTCCTAACACATATTCCAAAGAAAAATCTAAAGTCAGTCAATATTTTTTCGGTTTAAATACAACTTTACTCCCGAATTAATAAACTTTGATTACCTGGGTACWAATCCAGTTCACAAAACAAATTTTAAACTTTATTCAATTAAAATAAAAAATTAAAATATTTTACWAAATAAAATAAAAAATAAAAACACAAAAWTAAATTTACAACAWTTAGAGTTAAAAATTAAAATGGTTGCCGAYCTTCYGAACA	PP214459
33	*Acanthocheilonema reconditum*	12S ribosomal RNA	92	AGTCAACTCAATTGTTGTATATTTATTTTTGTGTTTTTTATTTTTTTATTTTATTTTTGTAAAATATTTTAATTTTTTATTTTTAATTGAATAAATGTTTAAAATTTGTTTTGTGAACTGGATTAGTACCCAGGTAATCAAAGTTTATTAATTCGGGAGTAAAGTTGTATTTAAACCGAAAAAATATTGACTGACTTTAGATTTTTCTTTGGAATATGTGTTAGGAGAGCCCTCCTTATTTGTTTAATTTTTTTTGGCACATGTATGATTGTTTTGTTATTATGTTATTTGTAATGCTTTAAACTTTTTTTGTTTTAAAACAGATATATATTTGGCTTATAGATTTTTTTTGCATGTATTACTATTGTTAATTTTCTTTGGATATTTTTTTTATTTTTTTTTGAAATTGGAAAAAAAAGTAATTTATTTTTTAGTGTTTTAGTGAATTTAATAAATAGAGTGGTACAAACACCCCCTCATAAGG	PP214460
39	*Acanthocheilonema reconditum*	12S ribosomal RNA	92	ACCGGTACTCTATTGTTATATTATTTAATTTTGTGTTTTTTATTTTTTTATTTTATTTTTGTAAAATATTTTAATTTTTTATTTTTAATTGAATAAATGTTTAAAATTTGTTTTGTGAACTGGATTAGTACCCAGGTAATCAAAGTTTATTAATTCGGGAGTAAAGTTGTATTTAAACCGAAAAAATATTGACTGACTTTAGATTTTTCTTTGGAATATGTGTTAGGAGAGCCCTCCTTATTTGTTTAATTTTTTTTGGCACATGTATGATTGTTTTGTTATTATGTTATTTGTAATGCTTTAAACTTTTTTTGTTTTAAAACAGATATATATTTGGCTTATAGATTTTTTTTGCATGTATTACTATTGTTAATTTTCTTTGGATATTTTTTTTATTTTTTTTTGAAATTGGAAAAAAAAGTAATTTATTTTTTAGTGTTTTAGTGAATTTAATAAATAGAGTGGTACAAACACCCCCTCTA	PP214461
41	*Acanthocheilonema reconditum*	12S ribosomal RNA	92	AAAAAACTCTACTTGTTATATTCGTTATTTTTAGTGTTTTTTATTTTTTTATTTTATTTTTGTAAAATATTTTAATTTTTTATTTTTAATTGAATAAATGTTTAAAATTTGTTTTGTGAACTGGATTAGTACCCAGGTAATCAAAGTTTATTAATTCGGGAGTAAAGTTGTATTTAAACCGAAAAAATATTGACTGACTTTAGATTTTTCTTTGGAATATGTGTTAGGAGAGCCCTCCTTATTTGTTTAATTTTTTTTTGGCACATGTATGATTGTTTTGTTATTATGTTATTTGTAAGGCTTTAAACTTTTTTTGTTTTAAAACAAATATATATTTGGCTTATAAATTTTTTTTGCATGTATTACTATTGTTAATTTTCTTTGGATATTTTTTTTATTTTTTTTTGAAATTGAAAAAAAAAGTAATTTATTTTTTAGGGTTTTAGGGAATTTAATAAATAAAGGGGTAAAAACCCCCCCCCAATAAAAA	PP214462
42	*Acanthocheilonema reconditum*	12S ribosomal RNA	100	TTTATTTTTTTATTTTATTTTTGTAAAATATTTTAATTTTTTATTTTTAATTGAATAAATGTTTAAAATTTGTTTTGTGAACTGGATTAGTACCCAGGTAATCAAAGTTTATTAATTCGGGAGTAAAGTTGTATTTAAACCGAAAAAATATTGACTGACTTTAGATTTTTCTTTGGAATATGTGTTAGGAGAGCCCTCCTTATTTGTTTAATTTTTTTTTGGCACATGTATGATTGTTTTGTTATTATGTTATTTGTAATGCTTTAAACTTTTTTTGTTTTAAAACAAATATATATTTGGCTTATAAATTTTTTTTGCATGTATTACTATTGTTAATTTTCTTTGGATATTTTTTTTATTTTTTTTTGAAATTGAAAAAAAAAGTAATTTATTTTTTAGGGTTTTAGGGAATTTAA	PP214463
45	*Acanthocheilonema reconditum*	12S ribosomal RNA	100	TTTTATTTTATTTTTGTAAAATATTTTAATTTTTTATTTTTAATTGAATAAATGTTTAAAATTTGTTTTGTGAACTGGATTAGTACCCAGGTAATCAAAGTTTATTAATTCGGGAGTAAAGTTGTATTTAAACCGAAAAAATATTGACTGACTTTAGATTTTTCTTTGGAATATGTGTTAGGAGAGCCCTCCTTATTTGTTTAATTTTTTTTTGGCCCATGTATGATTGTTTTGTTATTATGTTATTTGTAATGCTTTAAACTTTTTTTGTTTTAAAACAAATATATATTTGGCTTATAAATTTTTTTTGCATGTATTACTATTGTTAATTTTCTTTGGATATTTTTTTTATTTTTTTTTGAAATTGAAAAAAAAA	PP214464
49	*Acanthocheilonema reconditum*	12S ribosomal RNA	92	AACCTCTTCTATTGTTGTATATTTATTTTTGTGTTTTTTATTTTTTTATTTTATTTTTGTAAAATATTTTAATTTTTTATTTTTAATTGAATAAATGTTTAAAATTTGTTTTGTGAACTGGATTAGTACCCAGGTAATCAAAGTTTATTAATTCGGGAGTAAAGTTGTATTTAAACCGAAAAAATATTGACTGACTTTAGATTTTTCTTTGGAATATGTGTTAGGAGAGCCCTCCTTATTTGTTTAATTTTTTTTGGCACATGTATGATTGTTTTGTTATTATGTTATTTGTAATGCTTTAAACTTTTTTTGTTTTAAAACAGATATATATTTGGCTTATAGATTTTTTTTGCATGTATTACTATTGTTAATTTTCTTTGGATATTTTTTTTATTTTTTTTTGAAATTGGAAAAAAAAGTAATTTATTTTTTAGTGTTTTAGTGAATTTAATAAATAAAGTGGTACAAACCCCCCCCCCCAAAAAAA	PP214465
53	*Acanthocheilonema reconditum*	12S ribosomal RNA	92	TTTGGACTCTATTGTTGTAATATTTATTTTTGTGTTTTTTATTTTTTTATTTTATTTTTGTAAAATATTTTAATTTTTTATTTTTAATTGAATAAATGTTTAAAATTTGTTTTGTGAACTGGATTAGTACCCAGGTAATCAAAGTTTATTAATTCGGGAGTAAAGTTGTATTTAAACCGAAAAAATATTGACTGACTTTAGATTTTTCTTTGGAATATGTGTTAGGAGAGCCCTCCTTATTTGTTTAATTTTTTTTGGCACATGTATGATTGTTTTGTTATTATGTTATTTGTAATGCTTTAAACTTTTTTTGTTTTAAAACAGATATATATTTGGCTTATAGATTTTTTTTGCATGTATTACTATTGTTAATTTTCTTTGGATATTTTTTTTATTTTTTTTTGAAATTGGAAAAAAAAGTAATTTATTTTTTAGTGTTTTAGTGAATTTAATAAATAAAGGGGTACAAACACCCCCCCTCAAAAAAA	PP214466
55	*Acanthocheilonema reconditum*	12S ribosomal RNA	92	CCCTACGTCTCCTTGTTATAATATATATTTTTGTGTTTTTTATTTTTTTATTTTATTTTTGTAAAATATTTTAATTTTTTATTTTTAATTGAATAAATGTTTAAAATTTGTTTTGTGAACTGGATTAGTACCCAGGTAATCAAAGTTTATTAATTCGGGAGTAAAGTTGTATTTAAACCGAAAAAATATTGACTGACTTTAGATTTTTCTTTGGAATATGTGTTAGGAGAGCCCTCCTTATTTGTTTAATTTTTTTTGGCACATGTATGATTGTTTTGTTATTATGTTATTTGTAATGCTTTAAACTTTTTTTGTTTTAAAACAGATATATATTTGGCTTATAGATTTTTTTTGCATGTATTACTATTGTTAATTTTCTTTGGATATTTTTTTTATTTTTTTTTGAAATTGGAAAAGAAAGTAATTTATTTTTTAGTGTTTTAGTGAATTTAATAAATAGAGTGGTACAAACACCCCCTCATAAGG	PP214467
67	*Acanthocheilonema reconditum*	12S ribosomal RNA	90	ATCCTCTTGGTTCCGTCAGCCGTCGAGCTTCGTGTAGTACATTATTTTTACTTTATTTGTGCAAAATATTTTACTTTTTAACCTTCAATTGAATAAATGTTTAAAATTTGTTTTGTGAACTGGATTAGTACCCAGGTAATCAAAGTTTATTAATTCGGGAGTAAAGTTGTATTTAAACCGAAAAAATATTGACTGACTTTAGATTTTTCTTTGGAATATGTGTTAGGAGAGCCCTCCTTATTTGTTTAATTTTTTTTGGCCCCTGTATGAATGGTTTGTTATTATGTTATTTGGAATGCTTTAAACTTTTTTTGGTTTAAAACAAAAAAATATTTGGCTTAAAAATTTTTTTTGCCTGGATTACTATTGGTAATTTTCTTTGGAAATTTTTTTTATTTTTTTTTGAAATTGGAAAAAAAAGTAATTTATTTTTTAGGGGTTTAAGGAATTTAATAAATAGAGGGGGACAAACCCACCCCTCAAATAA	PP214468
74	*Acanthocheilonema reconditum*	12S ribosomal RNA	92	CGTTCGTTGCGCTGTAGCCGTATATCTTCTCTATAGTGTTTTTTATTTTTTTATTTTATTTTTGTAAAATATTTTAATTTTTTATTTTTAATTGAATAAATGTTTAAAATTTGTTTTGTGAACTGGATTAGTACCCAGGTAATCAAAGTTTATTAATTCGGGAGTAAAGTTGTATTTAAACCGAAAAAATATTGACTGACTTTAGATTTTTCTTTGGAATATGTGTTAGGAGAGCCCTCCTTATTTGTTTAATTTTTTTTGGCACATGTATGATTGTTTTGTTATTATGTTATTTGTAATGCTTTAAACTTTTTTTGTTTTAAAACAGATATATATTTGGCTTATAGATTTTTTTTGCATGTATTACTATTGTTAATTTTCTTTGGATATTTTTTTTATTTTTTTTTGAAATTGGAAAAGAAAGTAATTTATTTTTTAGTGTTTTAGTGAATTTAATAAATAGAGTGGTACAAACCTCCCCGTCAAATA	PP214469
78	*Dirofilaria immitis*	12S ribosomal RNA	95	ATACACTCATTTGTTGTATATTACGATTTTTTTTGTTTTTTTGTTTTATGTTTTTTTTTGTAAAATATTTAAATTTATTTATGTTTTTTTGTAATATTGAAAATTTGGTGTTTGAACTGGATTAGTACCCAGGTAATCAAAATTTATTAATTCGGGAGTAAAGTTTTGTTTAAACCGAAAAAATATTGACTGACTTTAGATTTTTCTTTGGAATATGTGTTTTTTTGGAGAGCCCTCTTTTATAGTGAATTTTGTTGGCGCATGTATGATTGTTTAGTTTTTACTTTTTTGGTAATGCTTTGTGTTTTATACATTTAAAACAGATATATATTTGGCTTATGGATTTATTTTTCATGTGTTACTATTGTTAATTTTCTTTGGATTAATTTTTAATTTTTTTGTTGAAATTGGAAAAGAAAGTAATTTTTTCTTAATGTAATAATGAATTTAATAAATAAAGTGGTACAACACTCCTTCATAAGGT	PP214470
83	*Acanthocheilonema reconditum*	12S ribosomal RNA	93	ATCGTCTCTATTGTTGTATATTTATTTTTGTGTTTTTTATTTTTTTATTTTATTTTTGTAAAATATTTTAATTTTTTATTTTTAATTGAATAAATGTTTAAAATTTGTTTTGTGAACTGGATTAGTACCCAGGTAATCAAAGTTTATTAATTCGGGAGTAAAGTTGTATTTAAACCGAAAAAATATTGACTGACTTTAGATTTTTCTTTGGAATATGTGTTAGGAGAGCCCTCCTTATTTGTTTAATTTTTTTTTGGCACATGTATGATTGTTTTGTTATTATGTTATTTGTAATGCTTTAAACTTTTTTTGTTTTAAAACAGATATATATTTGGCTTATAGATTTTTTTTGCATGTATTACTATTGTTAATTTTCTTTGGATATTTTTTTTATTTTTTTTTGAAATTGGAAAAAAAAGTAATTTATTTTTTAGGGTTTTAGTGAATTTAATAAATAAAGGGGTACAAACCCCCCCCCAAATAA	PP214470

### 2.3 Parasitological microscopic and immunocromatographic techniques

The blood samples from the dogs were collected in 1 mL K2 EDTA plastic microtubes and were maintained at 4°C. The Woo technique was used as a screening method to detect microfilaremic dogs (37). One-third of blue-line microhematocrit tubes were filled with whole blood, then sealed with plasticine, and centrifuged for 5 min at 11,000 rpm. Finally, they were observed under an optical microscope with a 10× objective, and their movement, which was either progressive rectilinear or non-progressive undulating, was recorded.

Microfilaremic samples were analyzed by the modified Knott technique (38). In brief, 1 mL of blood with K2 EDTA was mixed with 9 mL of 2% formalin and centrifuged at 1500 rpm for 5 min. After discarding the supernatant, 3 drops of methylene blue were added to the sediment. Afterward, 10 μL of this treated sample was spread on a slide and observed under a light microscope with a 40× objective. Microfilariae were identified by the following morphological criteria without sheath: a sharp cephalic end and a straight and sharp tail (*D. immitis*); a blunt cephalic end and a sharp and filiform tail, often ending in an umbrella hook (*D. repens*); and a blunt cephalic end with a prominent hook, and a flat and curved hooked tail (*A. reconditum*) (38–40). The microfilariae load in the positive samples was quantified in 20 μL of the sample that was diluted to 1:100.

Dog serum samples were tested for the presence of *D. immitis* antigens using a commercial immunochromatographic test kit (Uranotest Dirofilaria®, Urano Vet SL, Barcelona, Spain; sensitivity: 94.4%, specificity: 100%) according to the manufacturer’s instructions.

### 2.4 Molecular PCR endpoint detection

#### 2.4.1 DNA extraction

The commercial kit Corpogen^®^ DNA 2000 was used for the extraction of the genetic material following the manufacturer’s instructions. Briefly, 350 μL of each blood sample from microfilaremic dogs was processed. The samples were washed with a washing solution and columns and centrifuged at 12,000 rpm for 1 min four times. Then, 500 μL of lysis buffer and 50 μL of proteinase K were added. After 12 h of incubation at 56°C, they were vortexed and 500 μL of saline solution was added. They were then incubated on ice for 5 min and centrifuged at 12,000 rpm for 10 min. Then 600 μL of isopropanol was added, and the sample was shaken gently by inversion, centrifuged at 12,000 rpm for 5 min, and the supernatant was removed by inversion. The pellet was washed twice with 250 μL of 70% ethanol and centrifuged between washes at 12,000 rpm for 1 min. It was allowed to air dry for 20 min and finally the DNA was reconstituted with 100 μL of reconstitution solution for 1 h at 65°C. All solutions were provided by the manufacturer.

#### 2.4.2 *Dirofilaria immitis* and *Dirofilaria repens* multiplex PCR assay

The processed samples were subjected to multiplex PCR reactions for the detection of *D. immitis* and *D. repens* as per Gioia et al. (41). In brief, multiplex PCR reactions were performed in a SimpliAmp™ (Applied Biosystems™) using two sets of primers in the same mixture reaction. We used two general primer pair 12SF (5-GTTCCAGAATAATCGGCTA-3) and 12SRdeg (5-ATTGACGGATG(AG)TTTGTACC-3) and specific forward primer for *D. immitis* (12SF2B 5-TTTTTTTACTTTTTTTTTTGGTAATG-3) and a specific reverse primer for *D. repens* (12SR2 5-AAAAGCAACACACACAAATAA(CA)A-3) with an equimolar combination of general and specific primers in a single tube. The hybridization of these oligonucleotides amplifies the approximately 500 bp conserved region (12SF/12SRdeg) of filarials with a simultaneous amplification of the 204 bp *D. immitis* (12SF2B/12SRdeg) and/or 327 bp *D. repens* (12SF/12SR2) specific fragment. The final volume per reaction was 20 μL (1 μL genomic DNA, MgCl_2_, 1.5 mM, 0.2 mM dNTP, 0.5U Tucan Taq DNA polymerase (Corpogen), and 1 μM of each of the four primers) and the reaction had a thermal profile of 92°C for 1 min. Furthermore, 40 cycles at 92°C for 30 s, at 49°C for 45 s, at 72°C for 1 min, and final elongation step at 72°C for 10 min were performed. The amplification products were run on 2.5% ethidium bromide agarose gel at 95 V for 40 min followed by UV visualization. The specificity of the multiplex PCR assay for the two species was assessed by a control amplification of the DNA extracted from adult *D. immitis* and *D. repens* worms from the worm repository of the Zoonotic Diseases and One Health group of the University of Salamanca.

#### 2.4.3 *Acanthocheilonema reconditum* PCR assay

The PCRs were performed in a SimpliAmp™ (Applied Biosystems™) using a set of primers in the same mixture reaction. We used the general primer pair CxFrec (5′-GTGTTGAGGGACAGCCAGAATT-3′) and CXRrec (5′-GAACGTATATTCTGGATAGTGACCA-3′) previously designed on the COX1region. The sequences of the gene coding for COX1 of *A. reconditum* were obtained from GenBank (accession number MZ540221.1; MW656249.1; MW246127.1; MW138007.1; MT230063.1; MT193075.1; JF461456.1) and aligned using the online version of ClustalW2 (42).

The PCR was performed using an equimolar volume of primers (CxFrec/ CXRrec) in a single-tube reaction. The hybridization of these oligonucleotides amplifies the conserved region of approximately 118 bp (CxFrec/CXRrec). The final volume per reaction was 30 μL (3 μL genomic DNA, MgCl_2_, 1.5 mM, 0.2 mM dNTP, 0.5U Tucan Taq DNA polymerase (Corpogen), and 1 μM of each of the four primers). The thermal profile of the reaction was: at 95°C for 5 min; 40 cycles at 95°C for 60 s, at 50°C for 60 s, at 72°C for 30 s, and the final elongation step at 72°C for 5 min. The amplification products were run on 2.5% ethidium bromide agarose gel at 95 V for 40 min followed by UV visualization.

#### 2.4.4 Amplicon purification and DNA sequencing

The purification of the amplicon and sequencing was carried out following Gioia et al. (41) with some modifications. In brief, the specificity of the PCR amplification corresponding to *D. immitis* and *A. reconditum* on representative positive blood samples and to *D. immitis* and *D. repens* adult worms was assessed by amplicon purification followed by DNA sequencing. The species-specific amplicons were run on 2.5% ethidium bromide agarose gel followed by UV visualization. The concentration of the purified amplicons was spectrophotometrically measured using a ND-100 Spectrophotometer. The purified amplification products were then sequenced by SANGER sequencing (Macrogen Korea). The obtained sequences were aligned to the expected target sequences using the basic local alignment search tool (BLAST) (42).

The purified amplification products were then sequenced by SANGER sequencing (Macrogen Korea). The obtained sequences were aligned to the expected target sequences using BLAST (42) and should have close to 100% coverage (GenBank accession number KF707482.1, OR852434.1, MZ678927.1, OR854266.1, OR854267.1).

### 2.5 Statistical analysis

Data were analyzed using the SPCC 20.0 statistical program for Windows (SPSS Inc./IBM, Chicago, IL, United States). A descriptive analysis was carried out employing univariate analysis to determine the frequencies and bivariate analysis using chi-square test, from which a statistical analysis was carried out to determine the association between the variables. In all the cases, the significance level was set at *p* < 0.05.

Cohen’s Kappa coefficient, sensitivity and specificity were calculated to evaluate the efficacy of the diagnostic tests for the diagnosis of filariosis. The values obtained were classified as: coefficient < 0 (no agreement), between 0 and 0.19 (slight agreement), between 0.20 and 0.39 (fair agreement), between 0.40 and 0.59 (moderate agreement), 0.60 and 0.79 (substantial agreement), and 0.80 and 1.00 (almost perfect agreement).

## 3 Results

**Figure 2:**
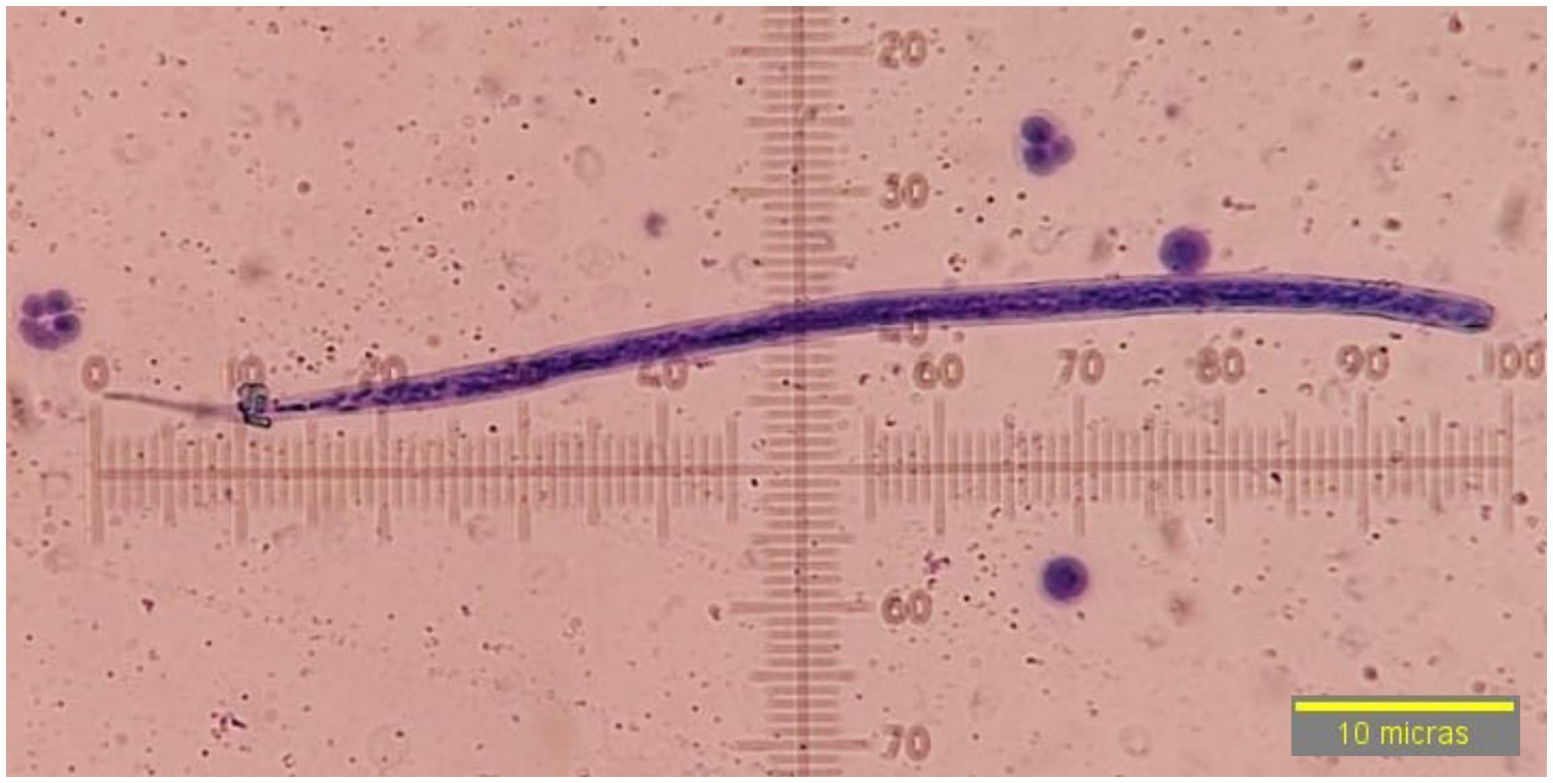
Microfilariae of *Dirofilaria immitis*, observed at 40× by modified Knott’s technique without sheath, sharp cephalic end, straight and sharp tail.

**Figure 3:**
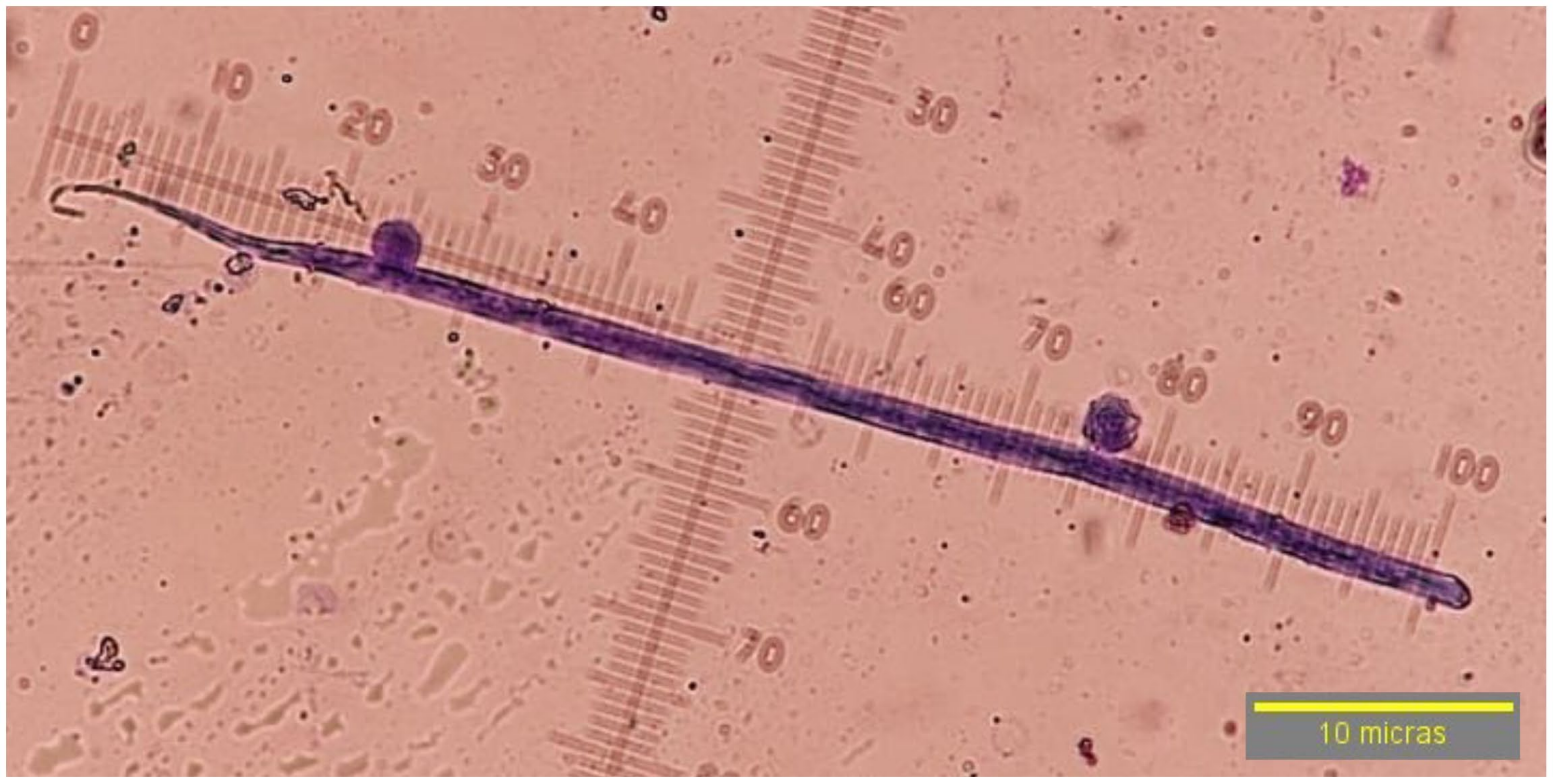
Microfilariae suggestive of *Dirofilaria repens*, observed at 40× by modified Knott’s technique without sheath, blunt cephalic end, sharp filiform tail, ending in an umbrella handle.

**Figure 4:**
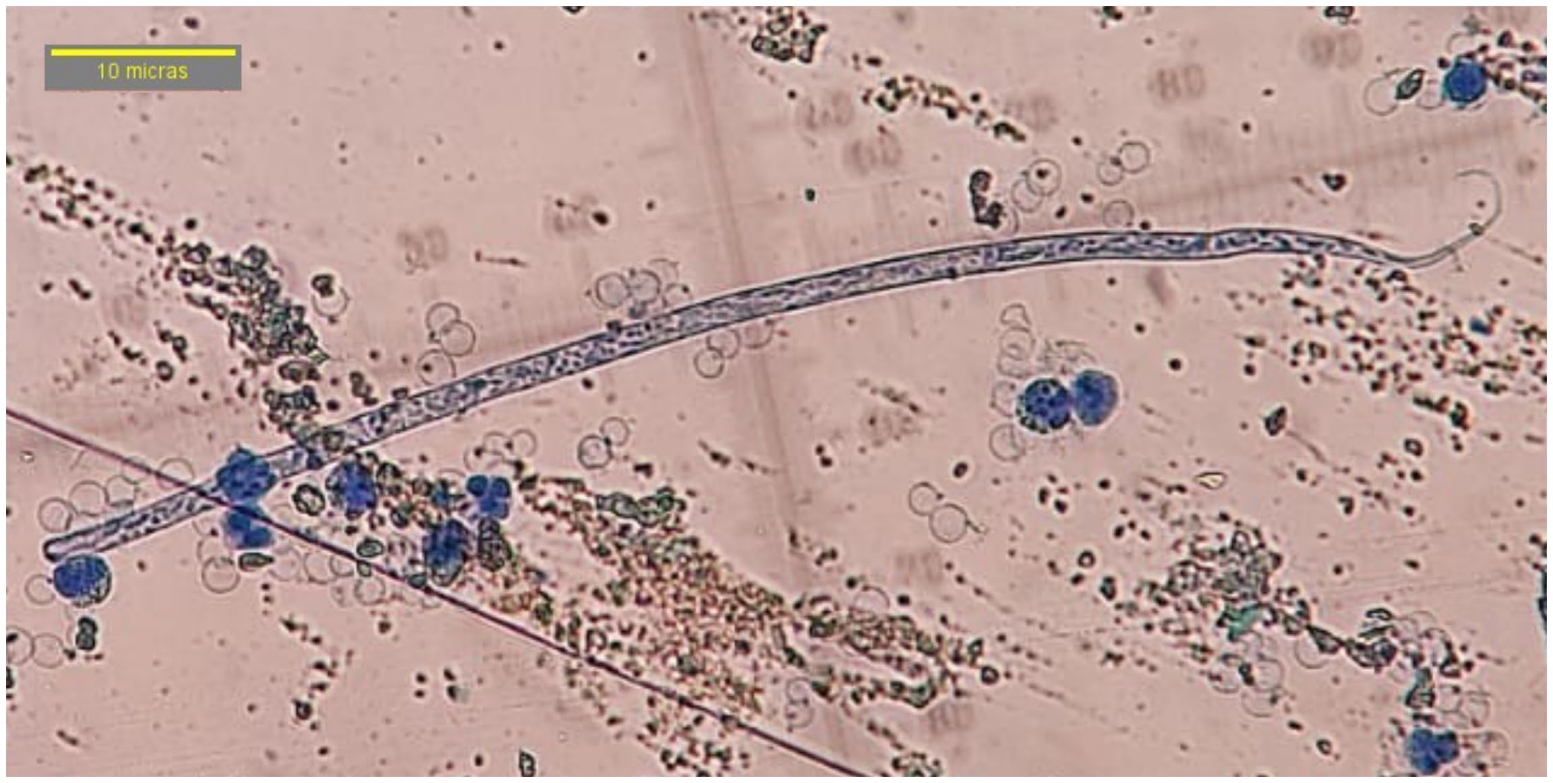
Microfilariae of *Acanthocheilonema reconditum*, observed in 40× by modified Knott’s technique without sheath, blunt cephalic end with prominent hook, flat and curved hooked tail.

Of the 400 dog samples tested, 75 had the presence of circulating microfilariae (18.75%) and 1.25% tested positive for the *D. immitis* antigen (5/400). In the 75 microfilaremics, progressive rectilinear movement was observed in 97.3% (73/75) of the samples and non-progressive undulant in 2.66% (2/75) of them. By morphology (head and tail), compatibility with *D. immitis* was seen in 4% of the microfilariae (3/75) that had a sharp cephalic end and a straight and sharp tail (Figure 2). Compatibility with *D. repens* was observed in 20% (15/75) of the samples characterized by a blunt cephalic end and a sharp and filiform tail, often ending in an umbrella hook (Figure 3). Compatibility with *A. reconditum* was noted in 76% (57/75) of the samples that featured a blunt cephalic end with a prominent hook and a flat and curved hooked tail (Figure 4).

Microfilaremic dogs samples were tested with D. immitis antigen test and 5/400 (1,25%) were positive. By PCR, 4/75 (5.3%) were positive for *D. immitis* and 71/75 (94.6%) for *A. reconditum*, with microfilaremic prevalences in relation to the total samples included in the study of 1% for *D. immitis* (4/400) and 17.25% for *A. reconditum* (71/400). Randomly, 27 fragments corresponding to 2 microfilaremic dogs with PCR product positive for *D. immitis* (2/4) and 25 for *A. reconditum* (25/71) were sequenced were sequenced by the Sanger technique (Macrogen, Korea) using primers 12SF and 12Rdeg for filarial generic, identifying the sequences as *D. immitis* with close to 100% query coverage and *A. reconditum* (Table 1). All microfilariae samples were negative for *D. repens*. One *D. immitis* antigen-positive dog showed microfilaremia and PCR-positive to *A. reconditum*. The Kappa index concordance for the results of the comparison of antigen testing for *D. immitis* with Woo test, morphological identification of *D. immitis* (Knott) with PCR *D. immitis* and morphological identification of *A. reconditum* (Knott) with *A. reconditum* PCR for the diagnosis of canine filariosis is shown in Table 2.

**Table 2:** Comparative assessment of the performance of the methods used in diagnostics in microfilaremic dogs according to Kappa index, confidence interval, sensitivity and specificity of the tests analyzed.

Test	Samples n/n total (%)	Kappa index	IC 95%	Sensitivity	Specificity
Ag Test Di × WOO	4/400 (1%)	0.078	0.038–0.118	80	82
Morfology Knott × PCR Di	4/75 (5.3%)	0.000	0.0–0.0	75	100
Morfology Knott × PCR Ar	57/75 (76%)	0.000	0.0–0.0	77	100

Ag, antigen; Ar: *Acanthocheilonema reconditum*; IC: confidence interval; Di: *Dirofilaria immitis*; PCR: Polymerase Chain Reaction.

All prevalences obtained by sex, age, municipality (Bucaramanga, Floridablanca, Girón and Piedecuesta), race, socioeconomic status and place of residence are shown in Table 3. Significant differences were found between the variables sex (*χ*^2^ = 6.57, df = 1, *p* < 0.01), municipalities (*χ*^2^ = 26,37, df = 7, *p* < 0.000071) and use of ectoparasiticides (*χ*^2^ = 11.93, df = 1, *p* < 0.002) for microfilaremic filariosis; the variables sex (*χ*^2^ = 6.32, df = 1, *p* < 0.012), municipalities (*χ*^2^ = 19.60, df = 3, *p* < 0.001) and use of ectoparasiticides (*χ*^2^ = 8.75, df = 1, *p* < 0.003) for *A. reconditum* and for dirofilariosis the only association was observed with use of ectoparasiticides (*χ*^2^ = 84.80, df = 1, *p* < 0.028) (Table 4).

**Table 3:** Filariosis prevalence in dogs in the metropolitan area of Bucaramanga by variables.

	Sample (n)	+ Microfilaremic	Prevalence mf	+ Ag Di test	Prevalence Ag Di	+ PCR Di	Prevalence PCR Di	+ PCR Ar	Prevalence PCR Ar
*Sex*									
Male	161 (40.3%)	40	24.84%	3	1.86%	2	1.24%	38	26.60%
Female	239 (59.8%)	35	14.64%	2	0.83%	2	0.83%	33	13.80%
*Age*									
1–2 years	113 (28.2%)	20	17.69%	1	0.88%	1	0.88%	19	16.81%
3–6 years	145 (36.3%)	26	17.93%	3	2.06%	2	1.37%	24	16.55%
>7 años	142 (35.5%)	29	20.42%	1	0.70%	1	0.70%	28	19.71%
*Breed*									
Mestize	313 (78.3%)	59	18.84%	4	1.27%	3	0.95%	56	17.89%
Pure	87 (21.8%)	16	18.39	1	1.14%	1	1.14%	15	17.24%
*Municipalities*									
Bucaramanga	145 (36.3%)	22	15.17%	2	1.37%	1	0.68%	21	14.48%
Floridablanca	74 (18.5%)	9	12.16%	1	1.37%	1	1.35%	8	10.18%
Girón	86 (21.5%)	31	36.04%	2	2.35%	2	2.35%	29	33.72%
Piedecuesta	95 (23.8%)	13	13.68%	0	0%	0	0%	13	13.68%
*Residential zone*									
Urban	206 (51.5%)	44	21.35%	4	1.94%	3	1.12%	41	19.90%
Rural	194 (48.5%)	31	15.97%	1	0.52%	1	0.51%	30	15.40%
*Socioeconomic level*									
Stratum 1	194 (48.5%)	33	17.01%	1	0.52%	1	0.51%	32	16.40%
Stratum 2	16 (4%)	2	12.50%	1	6.25%	1	6.25%	1	6.25%
Stratum 3	120 (30%)	28	23.33%	2	1.67%	2	1.66%	26	21.60%
Stratum 4	66 (16.5%)	12	18.18%	1	1.52%	0	0%	12	18.18%
Stratum 5	2 (0.5%)	0	0%	0	0%	0	0%	0	0%
Stratum 6	2 (0.5%)	0	0%	0	0%	0	0%	0	0%
*Place of permanence*									
Indoors	315 (78.8%)	62	19.68%	5	1.58%	4	1.26%	58	18.41%
Outdoors	85 (21.3%)	13	15.29%	0	0%	0	0%	13	15.29%
*Type of dwelling*									
House	363 (90.8%)	65	17.90%	4	1.10%	3	0.82%	62	17.07%
Apartment	37 (9.3%)	10	27.02%	1	2.70%	1	2.70%	9	24.32%
*Living with other animals*									
Dogs	185 (46.3%)	26	14.05%	1	0.54%	0	0%	26	14.04%
Cats	35 (8.8%)	9	25.17%	1	2.85%	1	2.85%	8	22.85%
Various species	180 (45%)	40	22.22%	3	1.66%	3	1.66%	37	20.50%
*Ectoparasiticides*									
Yes	293 (73.25%)	43	14.67	2	0.68%	1	0.34%	42	14.33%
No	107 (26.75%)	32	29.90%	3	2.83%	3	2.80%	29	27.10%
*Which?*									
Benzoylureas	1 (0.3%)	0	0%	0	0%	0	0%	0	0%
Fenilprazoles	7 (1.8%)	0	0%	0	0%	0	0%	0	0%
Isoxazolinas	135 (33.8%)	23	17.03%	1	0.74%	1	0.74%	22	16.26%
Various	150 (37.5%)	20	13.33%	1	0.66%	3	0.02	29	19.33%
None	107 (26.8%)	32	29.90%	3	2.80%	0	0%	20	19.69%
*Skin problems such as alopecia, nodules and scoriation.*									
Yes	100 (25%)	17	17%	0	0%	0	0%	17	17%
No	300 (75%)	58	19.33%	5	1.66%	4	1.33%	54	18%
Total	400	75	18.75%	5	1.25%	4	1.0%	71	17.75%

Ag: antigen; Ar: *Acanthocheilonema reconditum*; Di: *Dirofilaria immitis*; PCR: Polymerase Chain Reaction.

**Table 4:** Analysis of association of variables in dogs exposure to microfilaremic filariosis: *D. immitis* and *A. reconditum*.

**Variables**	**microfilaremic filariosis**			**Exposure to *D. immitis***			***A. reconditum***		
	**χ** ^2^	**df**	***p*<0.05**	**χ** ^2^	**df**	***p*<0.05**	**χ** ^2^	**df**	***p*<0.05**
Sex	6.57	1	0.01*	0.16	1	0.689	6.32	1	0.012*
Age	0,40	2	0.816	0.35	2	0.83	0.58	2	0.746
Municipalities	26.37	7	0.000071*	2.71	3	0.437	19.60	3	0.001*
Socioeconomic level	3.38	5	0.64	6.16	5	0.291	3.79	5	0.580
Residential zone	1.89	1	0.168	0.89	1	0.345	1.34	1	0.246
Type of dwelling	1.83	1	0.176	1.19	1	0.275	1.20	1	0.272
Place of residence	0.84	1	0.358	1.09	1	0.296	0.44	1	0.504
Ectoparasiticides	11.93	1	0.002*	4.80	1	0.028*	8.57	1	0.003*
Skin problems	0.26	1	0.605	1.34	1	0.24	0.05	1	0.821
Living with other animals	5.21	2	0.074	3.89	2	0.143	3.32	2	0.190

*p < 0.05.

## 4 Discussion

Filariae exhibit complex life cycles involving different hosts, as they develop through several larval stages. Considering that different specific vectors (mosquitoes, flies, ticks, fleas, and lice) are involved in their transmission to complete their biological cycle, the varying rates of infection, coinfection, and symptomatology, and the different treatments required to treat the infections caused by them, it is necessary to differentiate the various filariae species present in the dog for an accurate diagnosis of canine filariosis (2, 5, 43, 44). In our study, firstly, dogs with circulating microfilariae in the blood were identified by the Woo test, which was used as a screening test (45). Secondly, for the morphological identification of the species to determine the corresponding microfilariae, the Knott technique was used (10, 46–48). And, thirdly, to verify or elucidate the species of filaria that were visualized based on their morphological characteristics and when the result is not congruent and exact, as sometimes happens, due to the similarity between species and the strength of the smear, their molecular identification was carried out by PCR and the product was subsequently sequenced to identify the parasitic species (30, 49, 50).

The Woo and Knott techniques could generate false-negatives because of low parasitemia, interactions of a single sex of parasites, immaturity of the parasites, ectopic location of the adult worms, and application of microfilaricides, leading to a decrease in the sensitivity of the tests to identify the presence of filariae (46, 48–55). This is where the identification of antibodies and circulating filarial antigens in blood becomes important (9, 16, 40, 54, 56–58). Commercial tests for the detection of only circulating antigens of *D. immitis* in dogs and cats are available in Colombia, with sensitivities and specificities versus necropsy of over 94 and 100% respectively, allowing identification of dogs without microfilaremia and in which even a single adult worm is present (10, 28, 40). Even with high sensitivities and specificities, certain factors can affect the results, such as the age and sex of the parasite, the species of parasite other than *D. immitis*, not following the manufacturer’s instructions, or the use of lactones (8, 40, 49, 59). It is, therefore, necessary to develop and apply highly sensitive molecular methods based on single or multiple reactions for the identification of the genetic material of the different species present in the dogs at the time of veterinary consultation in order to establish an appropriate treatment protocol to treat the infections caused by each species of filaria (11, 40, 51, 60).

In our study, we observed microfilariae to be morphologically compatible with *D. immitis*, *D. repens*, and *A. reconditum*. However, PCR test and the subsequent sequencing of the specific amplified fragment ruled out the presence of *D. repens* and showed at least one dog with morphologically compatible microfilariae and testing positive for *D. immitis* antigen (gold standard for dirofilariosis), in which the sequencing after PCR also confirmed the presence of *A. reconditum*, thus pointing to the presence of a co-infected animal in this area. Similar findings have been reported by other authors (13, 41, 48, 50), recommending the use of PCR in veterinary clinics as a routine diagnostic method and in epidemiological studies wherever possible.

In our study, the integration of the methods used in the study such as WOO microscopy for the detection of microfilariae and species differentiation using PCR allowed the identification of the species for filariae present, which provide reliable data for clinical and epidemiological use.

In Colombia, the prevalence of *D. immitis* in dogs ranges from 0.91 to 53.2%, (22–27). The prevalence is 10.82% in Bucaramanga and the seroprevalence in humans is 6.71% (28). The prevalence varies between 4.81 and 61.3% for *A. reconditum* (13, 30) and the presence of *D. repens* is detected by morphological techniques in dogs that are put in shelters in Bucaramanga (29). In our study, the prevalence of *D. immitis* was 1% and that of *A. reconditum* was 17.75%, with one animal co-infected by both species, decreasing significantly from previously reported prevalences. This decrease could be due to the sampling performed. In the other studies, the samples used were mostly from uncontrolled stray dogs and dogs with owners with a poor socio-economic status, whereas in this study the dog samples came from veterinary clinics where the dogs underwent an annual check-up. The results observed for *A. reconditum* in dogs in this study represents the first report of the detection of this species in northeastern Colombia. In addition, we also found significant differences by socioeconomic level, which may be because of a non-uniform number of samples at all levels and variation in the administration of ectoparasiticides. A higher prevalence rate of parasitic infection was reported in animals in which ectoparasiticides were not administered, as other authors have reported (22, 27, 28, 46, 56, 57).

## 5 Conclusion

The use of highly sensitive diagnostic methods allows the identification of filarial species, leading to their classification and establishment of the appropriate treatment protocol to treat infections caused by each filarial species. Employing these methods also leads to generation of reliable data to be used at the clinical and epidemiological levels. The search for innovative diagnostic methodologies is fundamental to the development of veterinary care. In our study, we found, for the first time, the presence of a co-infected animal that tested positive for *D. immitis* antigen as well as for *A. reconditum* in the PCR microfilaremia in northeastern Colombia. It is therefore necessary to carry out a differential diagnosis of filariosis in dogs in this region and other nearby areas to improve the diagnosis and avoid clinical errors after treatment. Furthermore, taking into account that they are zoonotic diseases and that humans can be affected with a variety of symptoms and also become asymptomatic (silent infections), it is necessary to conduct epidemiological studies widely and improve the diagnosis of filariosis in order to control the disease more efficiently.

## Data availability statement

The datasets presented in this study can be found in online repositories. The names of the repository/repositories and accession number(s) can be found in the article/supplementary material.

## Ethics statement

The animal studies were approved by the Ethics Committee of the Cooperative University of Colombia (No. 006-2020). The studies were conducted in accordance with the local legislation and institutional requirements. Written informed consent was obtained from the owners of the animals for the participation of animals in this study.

## Author contributions

MVE-M: Conceptualization, Methodology, Writing – original draft, Writing – review & editing, Investigation, Resources, Validation. VHA-Q: Conceptualization, Funding acquisition, Methodology, Writing – review & editing, Writing – original draft, Resources, Validation. CRC: Methodology, Writing – review & editing, Data curation. JEJD: Data curation, Methodology, Writing – review & editing. MTO: Data curation, Methodology, Writing – review & editing. ADB: Data curation, Methodology, Writing – review & editing. MFC: Data curation, Methodology, Writing – review & editing. RM: Conceptualization, Methodology, Resources, Supervision, Validation, Writing – original draft, Writing – review & editing.
